# An Unusual Presentation of Juvenile Systemic Lupus Erythematosus

**DOI:** 10.7759/cureus.71301

**Published:** 2024-10-12

**Authors:** Sarah R Louis, Yash Nagpal, Charles H Hennekens, Jodi Fiedler

**Affiliations:** 1 Department of Pediatrics, Johns Hopkins University, Baltimore, USA; 2 Department of Pediatrics, Charles E. Schmidt College of Medicine, Florida Atlantic University, Boca Raton, USA; 3 Preventive Medicine, Department of Population Health and Social Medicine, Charles E. Schmidt College of Medicine, Florida Atlantic University, Boca Raton, USA; 4 Dermatology, Charles E. Schmidt College of Medicine, Florida Atlantic University, Boca Raton, USA

**Keywords:** cutaneous, dermatological, jsle, pediatrics, systemic lupus erythematosus

## Abstract

Systemic lupus erythematosus (SLE) is an autoimmune disease involving multiple organ systems that often mimic other conditions. The majority of patients with SLE show mucocutaneous manifestations, fatigue, fever, rheumatological manifestations, and weight loss as initial symptoms. SLE is classically managed medically with hydroxychloroquine, glucocorticoids, and/or immunosuppressives. Juvenile-onset systemic lupus erythematosus (jSLE) patients, who account for about 20% of SLE patients, tend to have earlier development of end-organ damage, and increased need for immunosuppressive therapies. We present a 12-year-old woman with a history of atopic conditions who initially presented for treatment of mild gastritis and a duodenal ulcer diagnosed by esophagogastroduodenoscopy (EGD). Several months later, the patient developed hematological abnormalities and rashes classically associated with SLE. Healthcare providers need to be aware of the distinct features of jSLE and modalities of treatment.

## Introduction

Systemic lupus erythematosus (SLE) is an autoimmune disease involving multiple organ systems that can mimic other conditions. Patients suffer from different manifestations of SLE that can be life-threatening, arising from hematologic, neuropsychiatric, renal, pulmonary, cardiovascular, and/or gastrointestinal complications. Juvenile SLE (jSLE) is more common in adolescent women than men [1-3]. The majority of patients with both SLE and jSLE show mucocutaneous manifestations, fatigue, fever, musculoskeletal manifestations, and weight loss as initial symptoms [4].

SLE and jSLE can be managed medically, and the drugs of proven efficacy include hydroxychloroquine, glucocorticoids, and/or immunosuppressive therapy. jSLE accounts for about 20% of all SLE patients. Patients with jSLE tend to have earlier development of end-organ damage which creates an increased need for immunosuppressive therapies [5].

The diagnosis and treatment of jSLE present many challenges to healthcare providers. The reasons include the variability of the presenting symptoms as well as the aggressive time course to end-organ damage [1]. The antinuclear antibody (ANA) test is an indicator of the underlying pathology and is the one most commonly performed when a health provider suspects SLE. Anti-double-stranded deoxyribonucleic acid (anti-dsDNA) for antigen-specific ANAs is also helpful to healthcare providers to confirm the diagnosis. In addition, low complement levels of C3 and C4 as well as an increased erythrocyte sedimentation rate (ESR) are also contributory to confirm the diagnosis. The medications commonly employed for SLE include hydroxychloroquine, glucocorticoids, and/or immunosuppressive therapies. The specific treatment used varies based on the diagnosis of jSLE or SLE as well as the individual manifestation of the condition [1]. Worldwide, increasing concerns about complications of jSLE, especially lupus nephritis led the Single Hub and Access point for pediatric Rheumatology in Europe (SHARE) initiative to develop recommendations for the diagnosis and management [6].

## Case presentation

A 12-year-old well-developed, well-nourished, and physically fit female adolescent developed heartburn, constipation, dull lower abdominal pain, nausea, and a low-grade fever. Past medical history included allergies to peanuts, tree nuts/pollen, asthma, and eczema. To manage these conditions, for about 2 years the patient was adhering to an inflammatory bowel disease diet. In addition, the patient was taking fluticasone propionate-salmeterol 250-500 mcg twice a day, lansoprazole 30 mg once a day, albuterol 90 mcg as needed, and montelukast 5 mg once a day. Pertinent negatives included no previous surgeries, no history of smoking, and no known past infection with Epstein-Barr virus and all immunizations were current. Esophagogastroduodenoscopy (EGD) showed mild gastritis and duodenal ulcer which was treated with sucralfate. Even after treatment, the patient had a persistent low-grade fever.

**Figure 1:**
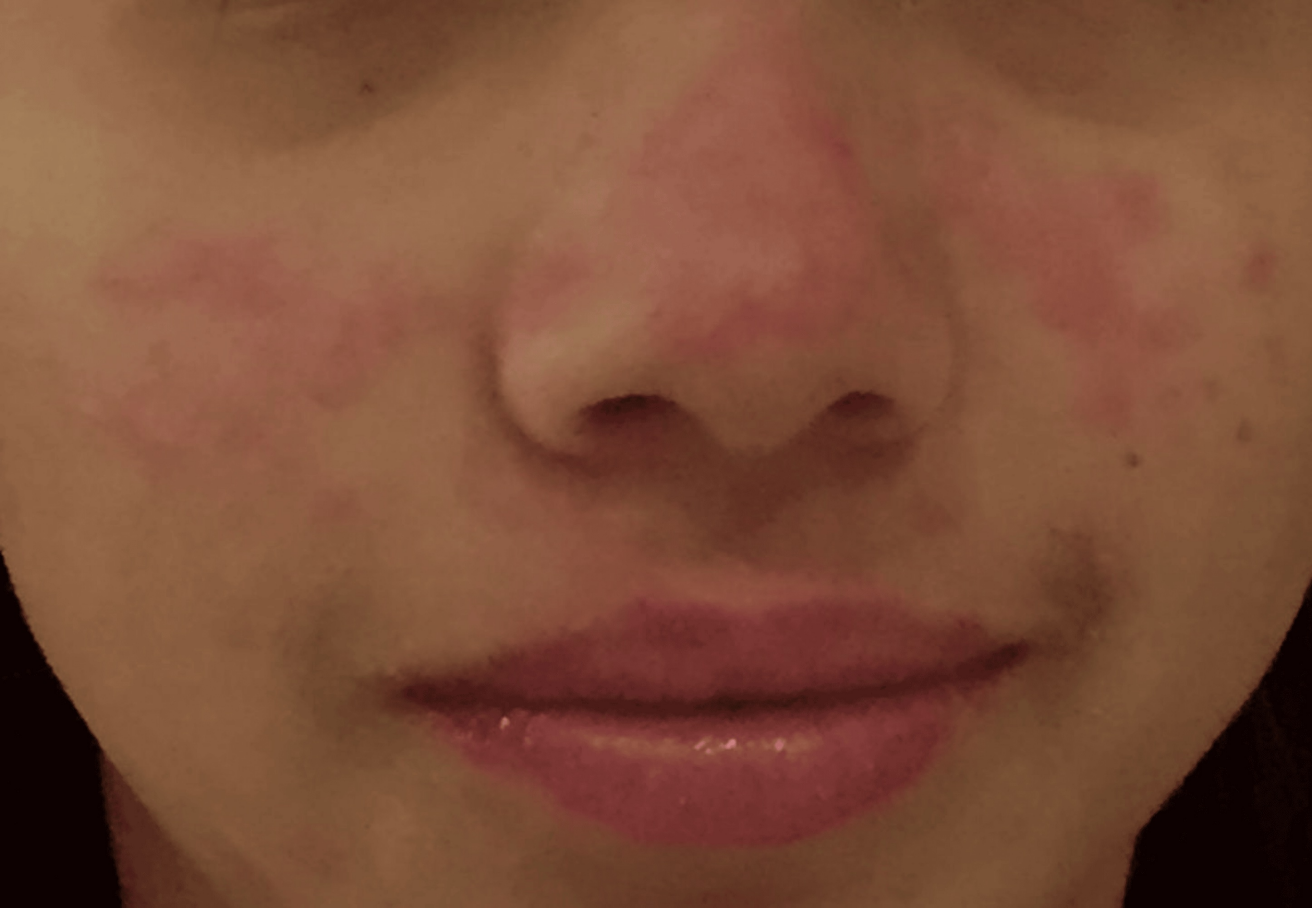
Malar rash on the second presentation to a healthcare provider

The then 13-year-old patient, presented to the rheumatologist with a malar rash (Figure 1) and receding hairline 2 weeks after its initial presentation.

Her blood tests showed high ESR, low white (WBC) and red (RBC) blood count (WBC), hemoglobin (HGB), hematocrit (HCT), C3 and C4 complement, elevated aspartate aminotransferase (AST), alanine transaminase (ALT), dsDNA 98, anti-ssDNA, high chromatin, ANA Screen A and B (Table 1). These results indicated that the patient had jSLE as there were abnormal ANA and dsDNA results that were consistent among SLE patients.

**Table 1:** Blood tests done on March 4, 2016 SED rate: sedimentation rate; WBC: white blood cell count; RBC: red blood count; HGB: hemoglobin; HCT: hematocrit; AST: aspartate aminotransferase; ALT: alanine transaminase; anti-dsDNA: anti-double-stranded deoxyribonucleic acid; anti-ssDNA: anti-single-stranded deoxyribonucleic acid; and ANA: antinuclear antibodies

Test	Result	Normal Range
SED rate	40 mm/h	0-20 mm/h
WBC	3.6 thousand/µL	4.2-10.0 thousand/µL
RBC	3.51 million/µL	4.20-5.40 million/µL
HGB	10.8 g/dL	12.0-16.0 g/dL
HCT	30.8%	37.0-47.0%
C3 complement level	19 mg/dL	90-207 mg/dL
C4 complement level	3 mg/dL	17-52 mg/dL
AST	52 U/L	<=37 U/L
ALT	47 U/L	<=78 U/L
Anti-dsDNA	98 IU/mL	<=40 IU/mL
Anti-ssDNA	1047.0 U/mL	<=99.0 U/mL
Chromatin	108.0 U/mL	<99.0 U/mL
ANA Screen A	45 U/mL	0-10 U/mL
ANA Screen B	23 U/mL	0-10 U/mL

The ANA was found to be high with a speckled (A) pattern which is found in SLE patients.

**Figure 2:**
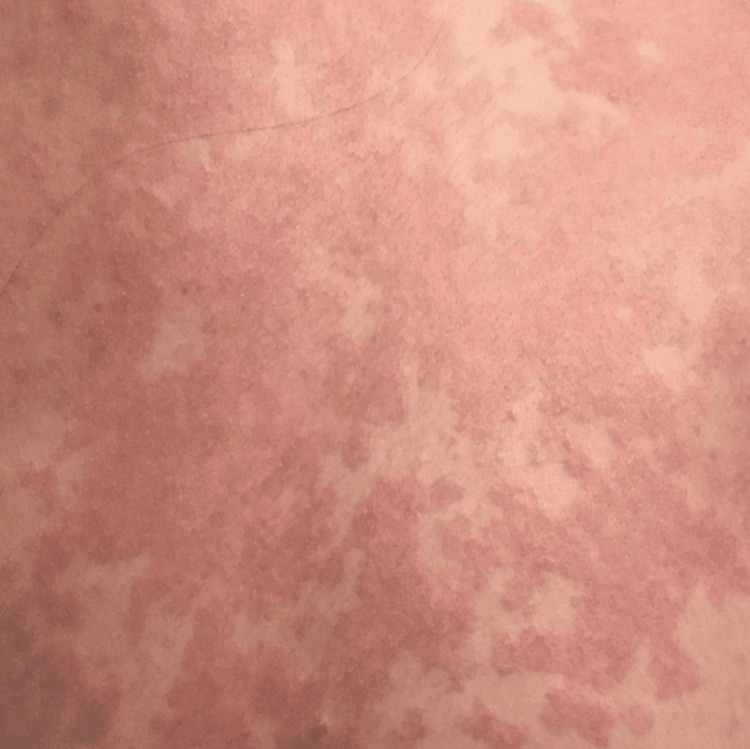
Mucocutaneous rash on April 9, 2016

By April 9, 2016, the patient had a mucocutaneous rash (Figure 2).

Direct immunofluorescence of a 0.3 cm punch skin biopsy was conducted when a patient came in with a mucocutaneous rash all over her body. The pathology report revealed a superficial perivascular and interstitial neutrophil-rich infiltrate mild with small vessel vasculitic changes and focal area of neutrophilic interface vacuolar changes as well as a positive direct immunofluorescence consistent with the diagnosis of SLE.

The rheumatologist diagnosed jSLE after the biopsy and blood panel was conducted and prescribed one daily dose of 200 mg hydroxychloroquine, 1000 mg of mycophenolate, and 20 mg of prednisone and avoidance of sunlight.

Two months later, the joint pain subsided, and the low-grade fever, malar, and mucocutaneous rash disappeared. She returned to her full functioning and her prednisone was tapered and discontinued but she continues hydroxychloroquine and mycophenolate.

On November 9, 2023, her most recent blood tests showed far more reassuring results, especially her ESR, C3, and C4 complement levels, AST, ALT, and WBC (Table 2).

**Table 2:** Blood tests on November 9, 2023 ESR: erythrocyte sedimentation rate; AST: aspartate aminotransferase; ALT: alanine transaminase; WBC: white blood cell count

Test	Result	Normal Range
ESR	35 mm/hr	4-25 mm/hr
C3 complement level	74.1 mg/dL	81.0-157.0 mg/dL
C4 complement level	19.24 mg/dL	13.00-39.00 mg/dL
AST	30 U/L	<=31 U/L
ALT	28 U/L	<=31U/L
WBC	3.91 K/cu mm	4.50-11.00 K/cu mm

**Figure 3:**
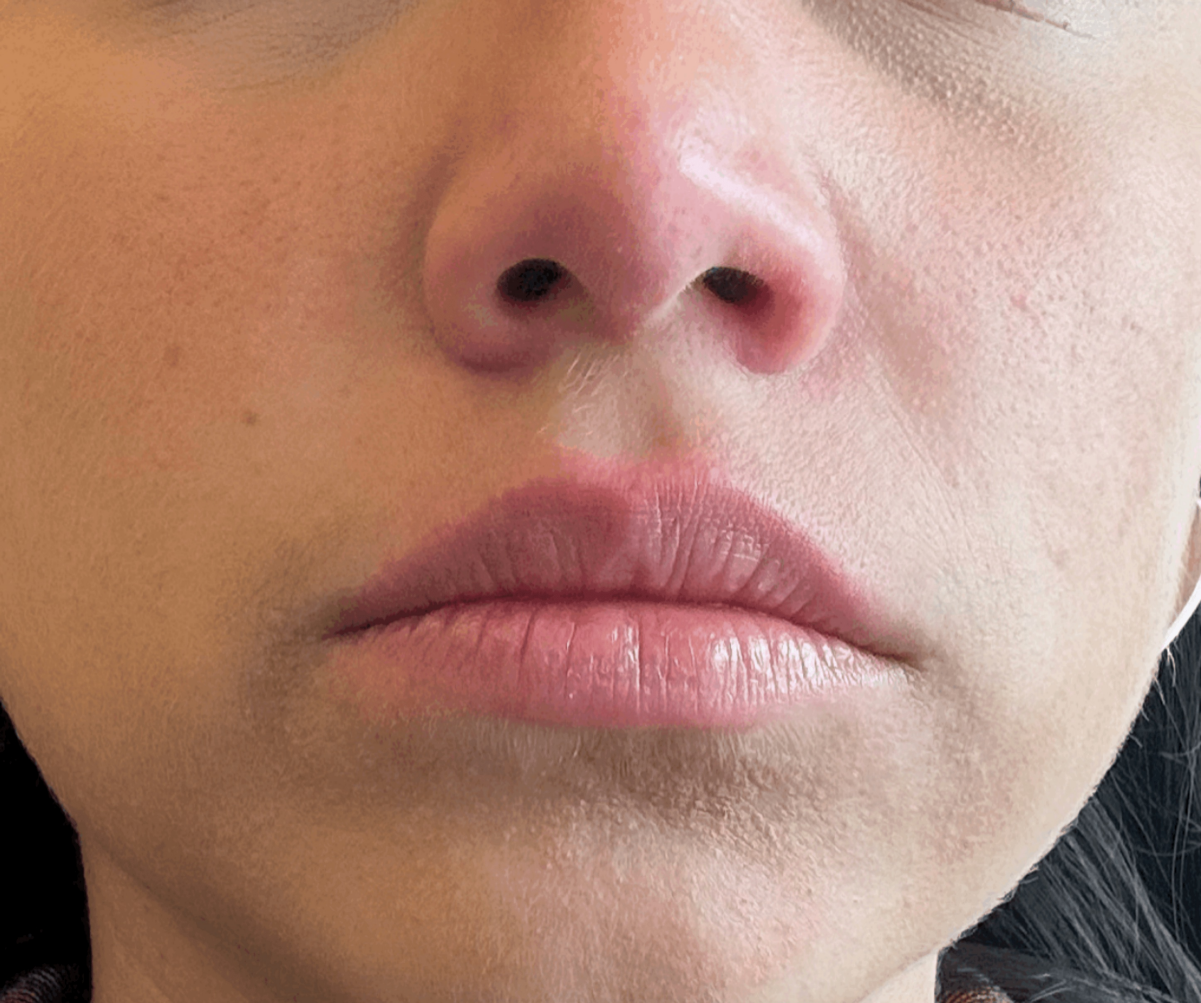
January 2024 image showing the disappearance of malar rash

In January 2024, the rashes had disappeared (Figure 3).

## Discussion

This patient originally presented solely with gastrointestinal symptoms and therefore underwent a full gastrointestinal workup including upper and lower endoscopy. Due to the nonspecific findings, the patient’s symptoms were treated individually. It was not until several months later that the patient was presented with the more classic symptoms of malar and mucocutaneous eruptions. At that time, the patient was referred to a rheumatologist and the diagnosis of jSLE was considered and appropriate treatment was initiated. Eight years later, this patient can flourish while attending university and participate in a variety of extracurricular activities. It is very common for jSLE patients to have different manifestations of the disease; therefore, it is not always easy to diagnose jSLE immediately. Patients differ in regard to case presentation, treatment response, and disease severity [1]. SLE is also often mistaken with other conditions which makes diagnosis even harder for the physicians. jSLE could range from mild disease presentation to life-threatening. The American College of Rheumatology criteria for SLE is typically used by physicians for diagnosis of jSLE, but jSLE patients will differ in which components of the criteria they will fall under at the time of diagnosis. Therefore, since the criterion for diagnosing jSLE is broad, it may take a while for physicians to accurately diagnose jSLE. Especially if a patient’s presentation of jSLE is abnormal [5]. As seen with our patient, initially presenting gastrointestinal issues that led to a suspicion of an autoimmune problem due to a presentation of a low-grade fever. In addition, studies have shown that there is an increased predisposition of jSLE reported among Asians, African Americans, Hispanics, and Native Americans [4]. The typical jSLE patients are adolescent females as well. The patient was prone to jSLE as she was Hispanic and a female. jSLE has an incidence of 0.3-0.9 per 100.000 children-years and a prevalence of 3.3-8.8 per 100.000 children-years which makes the disease rare among children [4]. Although jSLE is rare, it is still possible that a patient may have it. This is why it is important for physicians to never rule jSLE as a possibility especially if a patient is predisposed to getting the disease and showing symptoms of an autoimmune disorder.

Once a diagnosis is made it is important to minimize the disease activity within a patient, especially jSLE patients where lupus nephritis (LN) will present itself in 50-80% of jSLE patients causing renal damage [6]. jSLE patients are also more likely to be susceptible to liver disease and other organ damage. Typical medications given to jSLE patients include steroids, hydroxychloroquine, mycophenolate, azathioprine, and methotrexate. However, it is important to note that these medications do come with side effects [7]. New evidence shows long-term side effects of hydroxychloroquine such as retinopathy. It is important for patients on this drug to consider getting annual eye screenings [6]. As shown, our patient was treated with hydroxychloroquine, mycophenolate, and a steroid. The patient was advised to get an annual eye screening and a bone density exam while on her medications. Along with these medications it is typically advised for patients to wear sunblock as UV may trigger jSLE symptoms [7]. Lifestyle changes and medication management are crucial in mitigating the symptoms associated with jSLE with the goal of inducing remission. It is also important for jSLE patients to remain closely monitored during their course of treatment throughout their early years of treatment as jSLE patients are 18 times more likely to die than their peers with a standardized mortality ratio of 18.3 [7]. Existing literature commonly cites that jSLE is considered more severe when compared to adult-onset SLE. jSLE patients tend to have more severe organ damage and higher mortality rates in contrast to adult-onset SLE patients [6-8]. Additionally, jSLE is associated with a more abnormal presentation of early disease onset leading to poor prognosis with lower rates of ANA positivity and lower median anti-dsDNA titers [8]. However, when the ANA is positive among jSLE patients, this often coincides with renal, musculoskeletal, and hematological abnormalities. Our patient expressed musculoskeletal and hematological abnormalities alongside her positive ANA result as her disease activity was high at the time of diagnosis, common in jSLE patients [8]. Furthermore, it is especially important for jSLE patients to be diagnosed promptly while having a proper support system and caregivers to take care of the patient during their first presentation of the disease. Our patient was diagnosed and treated relatively quickly in order to reduce the disease activity which probably prevented her from experiencing life-threatening complications. A potential future consideration for healthcare providers to consider is the evolving generative AI, like ChatGPT, to personalize the care of the patient [9].

## Conclusions

The diagnosis and treatment of jSLE present many challenges to healthcare providers. The reasons include the variability of the presenting symptoms as well as the aggressive time course to end-organ damage. Adequately addressing the first requires a higher index of suspicion and the second, following the accurate diagnosis of jSLE, requires the earlier administration of immunosuppressive therapies. Once the patient is on immunosuppressive therapies and treatments, the goal is to ensure that their symptoms are treated promptly so that these jSLE patients can live a similar life to their peers. It is important to note that there is a need for expanded research efforts in the field of jSLE. A potential future consideration for healthcare providers to consider is the evolving generative AI, like ChatGPT, to personalize the care of the patient. As exemplified by this patient, teamwork in the diagnosis and treatment and a patient’s willingness to adhere to the lifestyle and adjunctive drug therapies have led to her leading a normal and productive life as a young adult.
